# Distribution of Informal Caregiving for Older Adults Living With or At Risk of Cognitive Decline Within and Beyond Family in Rural South Africa

**DOI:** 10.1093/geronb/gbaf008

**Published:** 2025-01-25

**Authors:** Sostina S Matina, Lenore Manderson, Michelle Brear, Farirai Rusere, F Xavier Gómez-Olivé, Kathleen Kahn, Guy Harling

**Affiliations:** MRC/Wits Rural Public Health and Health Transitions Research Unit, School of Public Health, University of the Witwatersrand, Johannesburg, South Africa; School of Public Health, University of the Witwatersrand, Johannesburg, South Africa; School of Social Sciences, Monash University, Melbourne, Australia; MRC/Wits Rural Public Health and Health Transitions Research Unit, School of Public Health, University of the Witwatersrand, Johannesburg, South Africa; School of Public Health and Preventive Medicine, Monash University, Melbourne, Australia; MRC/Wits Rural Public Health and Health Transitions Research Unit, School of Public Health, University of the Witwatersrand, Johannesburg, South Africa; School of Animal, Plant and Environmental Sciences, University of the Witwatersrand, Johannesburg, South Africa; MRC/Wits Rural Public Health and Health Transitions Research Unit, School of Public Health, University of the Witwatersrand, Johannesburg, South Africa; MRC/Wits Rural Public Health and Health Transitions Research Unit, School of Public Health, University of the Witwatersrand, Johannesburg, South Africa; MRC/Wits Rural Public Health and Health Transitions Research Unit, School of Public Health, University of the Witwatersrand, Johannesburg, South Africa; Institute for Global Health, University College London, London, UK; (Social Sciences Section)

**Keywords:** Familial care, Informal care, social networks

## Abstract

**Objectives:**

Aging populations will increasingly need care, much of this provided informally particularly in rural areas and in low and middle-income countries. In rural South Africa, formal support is severely limited, and adult children are frequently unavailable due to morbidity, early mortality, employment, and migration. We describe how care is shared within and between households.

**Methods:**

We conducted quantitative interviews with 1,012 household members and nonhousehold caregivers of 106 older adults (age ≥54) living with or at risk of cognitive decline in rural Mpumalanga, South Africa. Using descriptive statistics and regression analysis, we described how care is shared, with particular attention to generational patterns of care.

**Results:**

Spouses, the majority women, commonly considered themselves primary caregivers. informal care was spread among family, friends, and neighbors, most commonly by unemployed female relatives 1 or 2 generations younger than the recipient. A small number of paid caregivers, also mostly female, provided the most intensive care.

**Discussion:**

Informal care for older adults was spread widely, predominantly from coresident family but with important contributions from others. Family commitment to care reflected shared history, reciprocal relationships, and easy access to care tasks within the household. A deeper understanding of how informal care for older adults is shared is essential for developing targeted interventions.

## Background

With increased life expectancy in African countries, people are increasingly reporting chronic conditions significantly associated with older age (Guerchet et al., 2017; Olayinka & Mbuyi, 2014). In South Africa, older people experience multiple overlapping chronic health conditions. These include living with long-term HIV, and other infectious and noncommunicable diseases (NCDs) including hypertension, diabetes, and cancer, and vision and hearing impairments (Gómez-Olivé et al., 2017; Oni et al., 2014). The prevalence of cognitive decline and dementia is also rising in low- and middle-income countries (LMICs) as populations age (Hazzan et al., 2016; Mehta & Yeo, 2017). Although all older adults will require care at times, caregiving for a person with dementia is particularly demanding because cognitive decline reduces individuals’ ability to live independently and interact socially (Adedeji et al., 2022; Cao et al., 2020).

Dementia caregiving is commonly characterized as a dyadic process, with a single close family member providing near-continuous support. In practice, however, in both higher income and low-income settings, care is shared (Koehly et al., 2015; Spillman et al., 2019), reflecting the complexity of care needs involved and the capacities of different caregivers. In some high-income countries and settings, this informal care is buttressed by formal support (e.g., home visits by nurses) (Hajek et al., 2016), but in LMICs dementia-related and other caregiving largely falls on family members (Kane et al., 2021; Schatz, Ralston, et al., 2018). This reflects cultural norms and personal preferences for those living with dementia to be cared for in their own home or the home of the primary caregiver, and perceptions that children who send their parents to care homes are selfish (Agbawodikeizu et al., 2023; Rutagumirwa et al., 2020). Such care is provided informally and is unpaid; other female family members and friends of the care recipient may provide supplementary care or support (Jacobs et al., 2023; Roomaney et al., 2023). This trend is especially pronounced in rural areas, where access to formal care services is limited, but institutional settings or alternatives or supplements to informal care are limited country-wide. For example, the South African public health care system uses an Integrated Chronic Disease Management (ICDM) model which focuses on health promotion, self-management, population screening, facility reorganization, and clinical management support to meet population needs, but this does not include dementia-specific services (Godongwana et al., 2021).

The complex nature of households within and beyond households in southern Africa complicates who is available to provide care (Adamek et al., 2020; Nzima & Maharaj, 2020). This complexity includes missing generations and a growing proportion of grandparents who exclusively parent or coparent grandchildren, in the 1990s and early 2000s due to HIV mortality and increasingly when women move to find paid work or to study away from home (Roos & Wheeler, 2016; Schatz & Seeley, 2015; Schatz, Seeley, & Zalwango, 2018). These factors have resulted in “skipped generation” households, in which the only resident members are young dependents and older people, usually grandparents and grandchildren or great-grandchildren. Ideas of reciprocity and intergenerational obligation dictate that grandchildren care for their aging grandparents (Nzima & Maharaj, 2020; Schatz, Seeley, & Zalwango, 2018), although such ideas are not necessarily translated into practice. Moreover, many of these grandchildren (and great-grandchildren) are adolescents whose capacity to care may be limited by educational commitments, age, and experience (Kidman & Thurman, 2014; Stols et al., 2016). There is little evidence on how these factors lead to the spread of caregiving within and beyond households.

Most South African caregivers are women, reflecting strong patriarchal values and associated gender roles nationwide; women are responsible for domestic production, including maintenance of the house and yard or garden, for cooking and cleaning, and for caring for children and those who are ill or frail (Keahey, 2018). Moreover, a significant proportion of households are female-headed: nearly 46% in Mpumalanga province (Statistics South Africa, 2023, p. 3). Male involvement is limited by several factors: working-age men continue to routinely migrate from rural areas for work; remarriage after union dissolution is rare for women; and women often have children without formal (and culturally endorsed) commitment from the father—a minority South African births occur within coresidential partnerships (Bongaarts & Casterline, 2022; John & Nitsche, 2022).

Children reciprocate care as their parents age, with cultural norms of support extending to grandchildren so that they too contribute to caring for older adults. Children contribute in small ways to maintaining a household from a young age: contributing practically, emotionally, psychologically and financially (Mkhwanazi & Manderson, 2020, p. 194). This care work increases as senior household members age to include everyday labor (cooking, cleaning, assistance with everyday living) and managing ongoing and accumulating chronic health problems. Such efforts require the balancing of emerging chronic conditions in their elders and the growing demands of their immediate families (Mkhwanazi & Manderson, 2020, p. 156). Gender norms and the gendered composition and dynamics of households mean that provision of this care to older rural residents largely falls to women.

The majority of caregivers juggle multiple roles, including their own employment and childcare, so compounding stress and strain. This unequal caregiving is also likely to exacerbate existing gender disparities in chronic illness and multimorbidity among older South African women (National Department of Health (NDoH), 2019; Payne et al., 2017), given that caregivers of people living with dementia report a more significant subjective caregiving burden and lower quality of life than other caregivers (Karg et al., 2018). Addressing this issue requires systemic changes, better support systems, and resources to support female caregivers and to mitigate gendered inequities in health outcomes.

As suggested earlier, everyday care is generally provided in the care recipient’s home, in which case the primary caregiver may have moved residence, or in the caregiver’s home, in which case the care recipient may have been moved. However, LMIC households are frequently multigenerational—one-third of all South African households contained members at least two generations apart in 2011 (Makiwane, 2017)—and are strongly connected to nearby extended family and friends (Kelly et al., 2017). In such circumstances, despite a key person usually bearing responsibility for care, multiple people within a social network may contribute to caregiving, so limiting the workload for any one person (Mortazavi et al., 2015). A larger caregiving network reduces morbidity and mortality, and increases medication adherence for care recipients (Knight & Schatz, 2022; Naslund et al., 2020). A wider range of caregivers also benefits care recipients who thus receive both psychological and social support, so fostering a sense of belonging and mitigating loneliness, and instrumental support for functional limitations, physical care, and health care service access (Harling et al., 2020).

Understanding caregiver confidence in providing care to older adults is central to supporting caregivers in settings where formal care is lacking. Caregivers’ confidence is shaped by several factors, including their past experience, the support they receive, and their practical knowledge (Soroka et al., 2018). Confident caregivers enhance the wellbeing and functional outcomes of care recipients (Li & McLaughlin, 2012; Viinisalo, 1988), but little is known about confidence in capacity to care in rural LMIC settings. We therefore evaluated how informal care provision for over 100 older adults predicted to have or be at risk of cognitive impairment in rural South Africa was distributed across their social networks, with particular attention to generational and kinship patterns and numbers of caregivers.

Rural South Africa today is heterogenous in many ways, but is strongly influenced by Apartheid history (Collinson et al., 2007; Keahey, 2018; Madhavan & Schatz, 2007; Wittenberg & Collinson, 2020). Under this system, rural areas were allocated either for White or African residency, with the former consisting largely of farmland on which Africans might live and work with permits. The remaining 13% of land was allocated to one of 10 “Bantu homelands” linked to a specific nation and linguistic group (South Africa has 13 official languages, 10 belonging to the Bantu language group) to which Africans were often forced to relocate. These homelands were substantively under-resourced, leading to limited access to quality education, health care, and other service (Gaede & Versteeg, 2011; Spaull, 2013). Given limited infrastructure and transport options, daily life in rural areas continues to revolve around local villages despite many relatives working in distant urban centers (Ginsburg et al., 2021).

Our study was nested within the Agincourt Health and Socio-Demographic Surveillance System (HDSS) site of the South African Medical Research Council/University of the Witwatersrand Rural Public Health and Health Transitions Research Unit, in Bushbuckridge, Mpumalanga Province, South Africa. The Agincourt HDSS has maintained an annual census of ~120,000 people in 31 rural Mpumalanga villages since 1992 (Kahn et al., 2012). The area, formerly part of the Shangaan-language Gazankulu homeland, has limited employment opportunities in commercial food production and tourist venues proximate to Kruger National Park, driving circular labor migration to urban areas. The area also experienced significant numbers of deaths from HIV in the 1990s and 2000s. These two factors jointly result in relatively small cohorts of working age adults and many skipped-generation households—around 11% of households have skipped generations within Agincourt HDSS, of which two-thirds include adults aged over 60 (Schatz et al., 2015). Public health care services are limited to eight primary health care facilities that lack specialist services, and three district hospitals 25-60 kilometers away from the study villages and to which many residents cannot afford to travel.

## Methods

### Study Design

#### Participant Selection and Sampling Strategy

The Agincourt HDSS provides a robust platform, including an established community and rigorous sampling frame for numerous epidemiological studies. Our research was nested in HAALSI (Health and Aging in Africa: A Longitudinal Study of an INDEPTH Community in South Africa), a study undertaken as part of the wider Agincourt research program; it commenced in 2014 with a random sample of 5,059 adults aged ≥40 within the Agincourt HDSS area (Gómez-Olivé et al., 2018).

The HAALSI study is population-representative of older adults within the Agincourt site, and thus broadly representative of much of rural South Africa. The HAALSI Dementia Study began in 2019–2020 to estimate the prevalence and incidence of dementia and mild cognitive impairment (MCI) in the HAALSI cohort, with a second interview round in 2021 (see Bassil et al., 2022). We sampled index cases (older people with or at risk of cognitive impairment, hereafter referred to as care recipients) from Wave 2 of the HAALSI Dementia Study (see Manderson et al., 2022). We first included all participants clinically diagnosed with moderate or severe dementia in Dementia Study Wave 1 and who remained in the cohort at Wave 2. Because clinical diagnoses for Wave 2 were not available at the time of sampling, we identified additional potential index cases algorithmically, modeled on past work using similar methods to ascertain dementia status when clinical ratings are not available (Crimmins et al., 2011; Gianattasio et al., 2020). By sampling design, care recipients were approximately evenly split between men and women. We sampled individuals from those with the highest predicted dementia severity until we reached 116 index cases, a sample size calculated to be sufficient to power comparisons of meaningful differences (10 percentage points) in outcomes between equally sized groups (see Manderson et al., 2022). Ethnographic data collected in 21 of these households suggested that while those sampled did not all have an observable cognitive impairment, almost all received care daily (Brear et al., 2024).

For each care recipient, we first interviewed the designated primary household informant (typically the designated primary caregiver) from HAALSI Dementia Study Wave 2. They were asked to list all resident and nonresident household members plus any nonhousehold kin or nonkin who provided care to the care recipient. We then conducted quantitative interviews with all household members and caregivers who were not household members, aged ≥12, who provided verbal informed consent (with prior consent from parents or guardians for all minors). We included minors aged 12–17 because they are likely to play an important role in caregiving, and having a household member living with cognitive impairment is likely to have a substantial impact on their wellbeing. A total of 106 primary informant interviews and 1,012 respondent interviews were conducted.

Interviews were conducted face-to-face in Shangaan between July and December 2022 at respondents’ homes, with interviewers using tablet computers to capture data. The study received ethics approval from Wits Human Research Ethics Committee (Medical; M200373), University College London REC (152311/001) and Mpumalanga Province Health Research Ethics Committee, and letters of support from the Mpumalanga Department of Health and the Agincourt Community Advisory Group.

### Measures

#### Dependent variables

We investigated care distribution at both caregivers’ and care recipients’ level. Our primary outcome at the caregiver level was self-reported average weekly hours of care provided. We also calculated the percentage of all care to a given recipient provided by each caregiver, based on average weekly hours of care. Caregivers were asked if they considered themselves the primary caregiver to the care recipient; we additionally identified the individual(s) providing the most hours of care per week as an alternative measure of caregiving primacy. Finally, respondents were asked how confident they were in their capacity to provide care using a Likert scale (not at all confident; a little confident; somewhat confident; mostly confident; and extremely confident). These four measures reflect the overall burden on caregivers, the distribution of care responsibilities, self-perception of care roles, and caregiver self-efficacy.

At the care recipient level, we calculated total weekly hours of care received by summing across all caregivers as well as the maximum duration in years that the recipient had been receiving care from any current caregiver. Concentration of care was measured using the Herfindahl–Hirschman index (HHI), which assesses how a variable is distributed among distinct entities (Brezina et al., 2016; Rój, 2018). HHI is calculated as $\Sigma s_{i}^{2}$, where $s_{i}^{2}$ represents the share of the total amount each entity contributes and thus ranges from 0 to 1, with higher values indicating a greater concentration. We used reported weekly hours of caregiving from caregivers to calculate a care recipient-level measure of care concentration. These metrics provide insights into overall care needs, long-term care dynamics, and the distribution of care among caregivers.

#### Independent variables

We intentionally examined a wide range of independent variables to build a holistic view of how factors at multiple levels correlated with care provision. We considered care recipients’ gender, age in decades (<70, 70–80, 80–89, ≥90) and their predicted cognitive impairment severity (no dementia; mild dementia; moderate-severe dementia). We also considered caregivers’ age (<18, 19–39, 40–59,≥60), gender, marital status (married/coresident; never married; previously married), education (none; any primary, any secondary; any tertiary) and work status (fulltime work; part-time work; seeking work; out of workforce).

At the caregiver-care recipient dyad level, we considered the age difference between the care recipient and each caregiver in years, gender homophily (yes or no), relationship and coresidence. We categorized the relationship of caregiver to recipient at eight levels, five reflecting kinship and three others. Kin were distinguished as: spouse; nonspouse peer generation (sibling, cousin, in-law, etc.) or older generations; child; nonchild child generation (e.g., niece, nephew, daughter-in-law); grandchild and beyond (e.g., great niece, great-grandchild). Nonkin were separated into: employee/paid helper; friend; and neighbor.

### Statistical analyses

We first generated descriptive statistics for both care recipients and caregivers using median and interquartile range (IQR) for continuous variables and percentages for categorical variables. We assessed differences by gender in exposures and outcomes using Pearson’s $\chi^{2}$ tests for categorical variables and Wilcoxon rank-sum tests for continuous variables. We assessed care provision as experienced by the care recipient (i.e., total care per household) divided into eight relationship categories, and separately divided into three broader categories (resident relative; nonresident relative; nonrelative), using proportions of all care and 95% confidence intervals (CI). We further subdivided these analyses by gender of respondent. We calculated the age difference between potential caregivers and care recipients and presented this in histograms and violin plots, and calculated the gender homophily of care recipient-caregiver dyads.

To assess the correlates of caregiver provision level, we used negative binomial regression analysis for two highly right-skewed outcomes: (a) caregivers’ hours of care; and (b) caregivers’ proportion of all care to their care recipient—the latter to remove confounding of associations by the overall quantity of care provided to each recipient. We conducted binary logistic regression with outcomes of individuals being primary caregivers based on self-report and on weekly hours of care provided. Last, we ran an ordered logit regression with an outcome of reported confidence in providing care. All models were run as random intercept multilevel regressions (respondents nested in households), with covariates as listed in the exposures section. We used R version 4.3.2 for our statistical analyses (R Development Core Team, 2010).

## Results

Care recipients resided in 24 villages. Of the 116 care recipients whose caregivers were sampled, two had passed away, four were unlocatable, and the primary informants in four households declined to participate. Among the remaining 106 care recipients to whom a primary informant consented and was interviewed (Table 1), 1,020 household members and nonhousehold members who provided care to the recipients were named, and 1,012 of these subsequently consented and completed a survey (three out-migrated, one died, three were noncontactable, and one declined). By design the sample was approximately balanced on gender. Over half of the care recipients were over 80 years of age. Three-quarters of female care recipients (76%) were widowed, whereas nearly half of male recipients (49%) were married. Reflecting the larger numbers of women predicted to have poor cognition in the HAALSI Dementia Study, female care recipients were more likely to be predicted to have dementia. Less than 20% of respondents reported any formal help caring for the care recipient; with no-one in 63% of care recipient networks reporting any help. Most reported help (76%) was from the Department of Health, which included clinic visits. Thirty-two of the 106 care recipients, the majority men, received care from spouses.

**Table 1.** Care Recipient Characteristics

Characteristic	Overall (*n* = 106)	Male (*n* = 51)	Female (*n* = 55)	*p*-Value ^a^
	%	%	%	
Marital status				<.001
Married/coresident	29.5	49.0	11.1	
Never married	4.8	3.9	5.6	
Divorced	8.6	9.8	7.4	
Widowed	57.1	37.3	75.9	
Predicted dementia				<.001
No dementia	15.1	31.4	0.0	
Mild dementia	67.0	58.8	74.5	
Moderate–severe dementia	17.9	9.8	25.5	
Age group				.80
Under 70	14.2	15.7	12.7	
70–79	24.5	27.5	21.8	
80–89	43.4	41.2	45.5	
90+	17.9	15.7	20.0	
Receiving old people’s grant	16.0	9.8	21.8	.12

^a^Pearson’s chi-squared test for categorical variables; Wilcoxon rank sum test for continuous variables.

**Figure 1.**
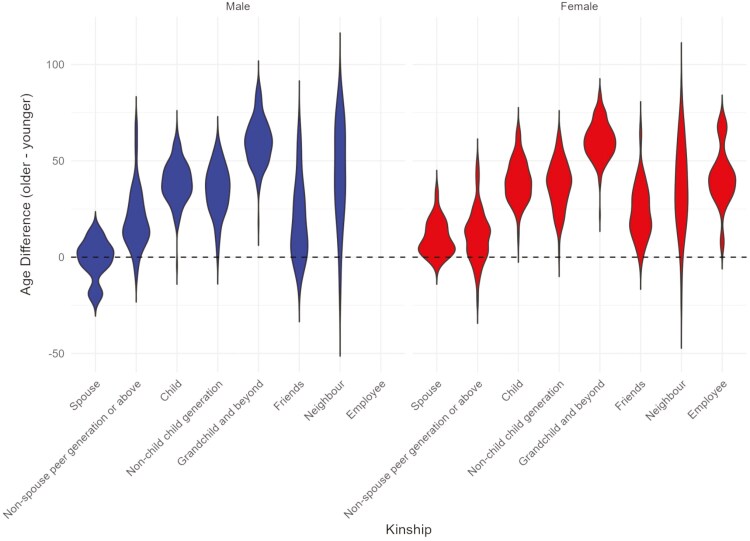
Age difference between caregiver and care recipient by relationship type and caregiver gender.

Table 2 presents characteristics of the caregivers. Almost two-thirds of reported caregivers were female. Most respondents were aged under 40 (59% of men, 48% of women), whereas 14% were aged under 18. The median age difference between caregivers and care recipients was 42 years (IQR: 29, 57); almost 20% of respondents over 40 years were younger and very few older than the care recipient (detail provided in Supplementary Figure 1). Figure 1 presents the distribution of age differences between the care recipient and caregivers—larger values showing the recipient being older than the caregiver. When stratified by relationship type, beside expected generational kin differences, almost all nonkin paid caregivers were female and around 40 years younger than their care recipients. Friends were typically closer in age than neighbors to the care recipient.

**Table 2.** Respondents Characteristics

Characteristic	Overall (*N *= 1,102)		Male (*n* = 398)		Female (*n* = 614)		*p*-Value ^a^
	%	Median (IQR)	%	Median (IQR)	%	Median (IQR)	
Caregiver age							.007
Under 18	13.8		14.1		13.7		
19–39	38.0		43.7		34.4		
40–59	30.6		27.6		32.6		
60+	17.5		14.6		19.4		
Marital status							<.001
Married/coresident	36.5		38.9		34.9		
Never married	42.5		48.2		38.8		
Previously married	21.0		12.8		26.4		
Number of living children		2 (0, 4)		2 (0, 3)		2 (1, 4)	.001
Work status							<.001
Fulltime work	19.8		27.6		14.7		
Part-time work	9.6		11.8		8.1		
Seeking work	22.5		19.1		24.8		
Out of workforce	48.1		41.5		52.4		
Age difference		42 (29, 56)		45 (32, 58)		41 (27, 55)	.003
Gender homophily							<.001
Both female	30.2				49.8		
Male recipient, female provider	30.4				50.2		
Female recipient, male provider	20.5		52.0				
Both male	18.9		48.0				
Relation status							<.001
Spouse	3.3		1.5		4.4		
Kin nonspouse peer generation or above	9.3		8.5		9.8		
Child	28.0		32.2		26.4		
Kin nonchild child generation	13.5		10.3		15.6		
Grandchild and beyond kin	30.7		36.2		27.2		
Friends	4.5		3.8		5.0		
Neighbor	7.7		6.5		8.5		
Employee	1.9		0.3		2.9		
Resident status							.008
Coresident	34.3		38.4		31.6		
nonresident	51.4		51.0		51.7		
Nonrelative	14.2		10.6		16.6		
Education							<.001
None	13.5		8.5		16.8		
Primary education	19.8		20.1		19.5		
Secondary education	56.5		58.5		55.2		
Tertiary education	10.2		12.8		8.5		

*Notes*: IQR = interquartile range.

^a^Pearson’s chi-squared test for categorical variables; Wilcoxon rank sum test for continuous variables.

## Provision of Caregiving

All but seven respondents reported providing care of some kind, with 783 (77%) reporting at least one hour per week (86% of coresidents, 73% of nonresidents). Respondents’ marital status varied, reflecting the wide range of life stages represented in the sample, with men more likely to be never or currently married and women more likely to be previously married (separated, divorced or widowed). Fewer than 30% of caregivers were working despite more than 60% of respondents being of working age; almost half of respondents were out of work and not looking for a job. Figures for gender homophily suggested no preferential involvement in care based on the gender of the recipient.

**Figure 2.**
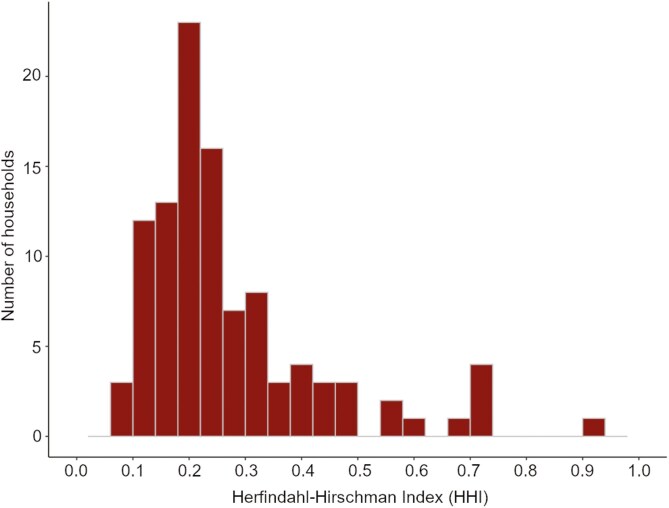
Concentration of care provision to care recipients.

The average care recipient received 52 hours of care weekly (IQR: 24, 70). Figure 2 shows that the concentration of care was generally low but varied widely across the 106 households, with a median HHI of 0.22 (IQR: 0.18, 0.31). Only 10 households had an HHI over 0.5, that is, one individual provided more than 70% of all care hours. Caregivers had provided care for a median of five years (IQR: 3, 10). The median longest care dyad per household was 13 years (IQR: 7, 20), and the longest dyadic care relationship was 40 years.

Alt text: Histogram showing most households have Herfindahl–Hirschman indices under 0.3 with a mode of 0.2. A small minority of households have higher values with one household only over 0.75.

**Figure 3.**
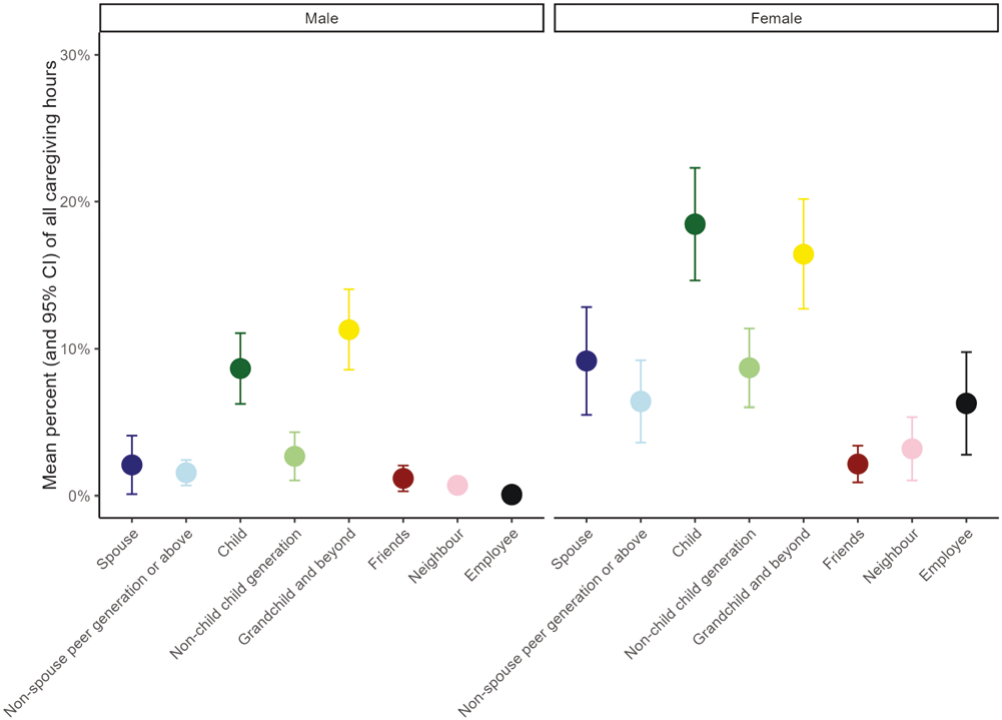
Distribution of caregivers’ hours from care recipient’s point of view based on relationship type and caregiver gender. CI = confidence interval.

Figure 3 shows how care was shared proportionally among kin and nonkin caregivers by caregiver gender (Supplementary Table 1 provides these values in absolute hours per provider, Supplementary Figure 2 shows per-recipient composition by relationship type). On average, respondents reported 5.5 hours of care per week. Female spouses and daughters provided a substantially greater proportion of care than their male counterparts, even allowing for a greater number of female caregivers. The most common caregivers were adult children (25% of all caregivers) who each provided a mean of 5.1 hours of care per week, or 13.9 hours per care recipient in total. Grandchildren provided a similar amount of care, comprising 23% of all caregivers and providing a mean of 5.6 hours of care a week each, or 16.6 hours per care recipient in total. However, when present in the caregiving network, paid nonkin (21.9 hours) and spousal (18.1 hours) caregivers provided more care per week on average than individual children and grandchildren.

When caregivers were stratified by relative and residence status, coresident kin provided the greatest proportion of all care, followed by nonresident kin and then nonkin; female respondents in all groups reported providing more hours of care each week than male respondents (Supplementary Figure 3). Supplementary Table 2 describes all dependent caregiving variables by caregiver gender. Female respondents reported higher involvement in primary caregiving, both in terms of care hours (15.8% vs 5.8%) and self-reported caregiving (33.4% vs 22.5%), and in providing more weekly hours of care (median 3 vs 2), although they were no more confident of their caregiving.

### Correlates of Caregiving

Table 3 provides results from a multivariable multilevel model of absolute care hours provided, both as rate ratios and difference in marginal mean hours. Household residents provided significantly more hours of care (incidence rate ratio [IRR]: 2.17, 95%CI: 1.58–2.75, or 4.7 more hours of weekly care) than nonresidents. Respondents aged over 60 provided the most hours of care, and those aged 19–39 the fewest. Employment was negatively associated with care provision—those seeking work provided around twice as much care as those in full-time work. Levels of care provision were higher both from female respondents and to female care recipients—the highest care levels were seen when both individuals were female. Each decade of age difference between caregiver and recipient was associated with an approximately 10% decrease in both hours and percentage of care provided. Female caregivers caring for female older adults provided notably more care than any other gender pairing. As shown in descriptive analysis, employees and spouses provided the most intensive care, whereas neighbors, same-generation kin and friends provided the least. In the case of employees this amounted to almost 10 additional hours of care relative to spouses, and even more compared to other respondents.

**Table 3.** Multilevel Multivariable Negative Binomial Regression Models to Predict Hours of Care Provided

Variable	Ratio	Difference
Coresident (vs nonresident)	2.17 [1.58, 2.75]	4.66 [2.82, 6.50]
Caregiver age (vs ≥60)		
<18	0.88 [0.15, 1.61]	−0.68 [−4.97, 3.62]
19–39	0.60 [0.20, 1.01]	−2.77 [−6.49, 0.95]
40–59	0.72 [0.42, 1.01]	−1.80 [−3.94, 0.35]
Marital status (vs married)		
Never married	1.06 [0.77, 1.35]	0.34 [−1.21, 1.90]
Previously married	0.88 [0.68, 1.07]	−0.73 [−1.92, 0.46]
Work status (vs full-time work)		
Part-time work	1.00 [0.68, 1.32]	−0.02 [−1.84, 1.80]
Seeking work	2.00 [1.41, 2.59]	4.78 [2.19, 7.38]
Out of workforce	0.89 [0.60, 1.18]	−0.68 [−2.53, 1.17]
Gender homophily (vs both male)		
Male recipient, female provider	1.24 [0.94, 1.54]	1.27 [−0.24, 2.78]
Female recipient, male provider	1.18 [0.74, 1.63]	1.02 [−1.39, 3.44]
Both female	1.88 [1.16, 2.60]	4.08 [1.16, 7.00]
Age difference (per decade)	0.94 [0.80, 1.08]	−0.36 [−1.23, 0.51]
Relationship type (vs spouse)		
Nonspouse peer generation or above	0.43 [0.22, 0.64]	3.51 [−5.04, −1.98]
Child	0.66 [0.32, 1.00]	−2.21 [−4.80, 0.39]
Nonchild child generation	0.64 [0.30, 0.99]	−2.13 [−4.37, 0.11]
Grandchild and beyond	0.81 [0.27, 1.35]	−1.12 [−4.64, 2.39]
Friends	0.52 [0.21, 0.83]	−2.81 [−4.76, −0.87]
Neighbor	0.36 [0.11, 0.60]	−3.88 [−5.59, −2.16]
Employee	2.76 [0.84, 4.68]	9.55 [−0.65, 19.75]
Education (vs primary)		
No education	1.47 [0.87, 2.07]	2.45 [−0.50, 5.41]
Secondary education	0.84 [0.65, 1.03]	−1.00 [−2.29, 0.29]
Tertiary education	0.84 [0.51, 1.17]	−0.91 [−2.83, 1.02]
Care recipient predicted dementia (vs no dementia)		
Mild dementia	0.63 [0.33, 0.93]	−2.82 [−6.07, 0.42]
Moderate to severe dementia	0.86 [0.42, 1.31]	−0.82 [−3.57, 1.93]

*Notes*: The first column shows ratios of hours compared to the base category and 95% confidence intervals (CI), whereas the second column shows differences in marginal mean hours and 95% CI.

Supplementary Table 3 shows results from a multivariable model of the proportion of all care to a care recipient provided by each caregiver. Results were broadly similar to those from models of absolute hours of care, but differences by age were less pronounced, implying that older respondents were in households with more overall care provision.

Table 4 provides results from multivariable multilevel models for the other three caregiving outcomes. Household residents were substantially more likely to report that they were primary caregivers compared to nonresidents (odds ratio [OR]: 3.69; 95%CI: 2.28–5.96), as were those closer in age to recipients, female caregivers, and those with tertiary education. These patterns were largely repeated for those who were primary caregivers by hours provided, although the associations with household residency and female gender were stronger, as was the association between fulltime employment and less caregiving primacy. Caregiver confidence was higher for those with tertiary education (OR: 2.69; 95%CI: 1.45–4.98), those who had been caregivers for longer and those who provided more hours of care per week; in contrast it was lower for those out of the workforce. Confidence was not associated with respondents’ age or sex.

**Table 4.** Multivariable Multilevel Binary and Ordinal Logistic Regression Models to Predict Primary Caregiving Status and Confidence to Care

Variable	Self-reported primary caregiver ^a^	Most hours of caregiving ^a^	Confidence in ability to care ^b^
Coresident (vs nonresident)	3.69 [2.28, 5.96]	5.43 [2.56, 11.5]	0.99 [0.70, 1.40]
Age difference (per decade)	0.79 [0.66, 0.94]	0.74 [0.66, 0.94]	1.05 [0.91, 1.21]
Marital status (vs married)			
Never married	0.67 [0.40, 1.14]	1.46 [0.59, 3.56]	0.87 [0.57, 1.34]
Previously married	0.72 [0.44, 1.21]	0.69 [0.28, 1.67]	1.10 [0.72, 1.67]
Work status (vs full-time work)			
Part-time work	0.92 [0.45, 1.87]	5.06 [1.40, 18.2]	0.77 [0.43, 1.37]
Seeking work	0.83 [0.47, 1.46]	4.07 [1.34, 12.3]	0.87 [0.55, 1.38]
Out of workforce	0.57 [0.33, 0.97]	1.53 [0.53, 4.45]	0.44 [0.28, 0.69]
Number of living children	1.00 [0.98, 1.01]	1.00 [0.85, 1.17]	1.02 [0.94, 1.10]
Education (vs primary)			
No education	1.13 [0.57, 2.19]	0.37 [0.12, 1.10]	1.03 [0.60, 1.76]
Secondary education	1.00 [0.59, 1.68]	0.43 [0.20, 0.95]	1.12 [0.75, 1.68]
Tertiary education	1.98 [0.95, 4.09]	0.16 [0.03, 0.84]	2.69 [1.45, 4.98]
Average weekly care provision to recipient (hours)	1.13 [1.10, 1.16]	1.30 [1.10, 1.15]	1.07 [1.04, 1.09]
Length of care (years)	1.07 [0.92, 0.99]	0.94 [0.90, 1.00]	1.04 [1.01, 1.07]
Caregiver age (per decade)	0.96 [0.70, 1.32]	1.03 [0.98, 1.08]	1.00 [0.94, 1.00]
Care recipient predicted dementia (vs no dementia)			
Mild dementia	1.07 [0.51, 2.26]	1.29 [0.43, 3.85]	1.40 [0.70, 2.79]
Moderate to severe dementia	0.73 [0.28, 1.91]	1.30 [0.79, 2.13]	1.11 [0.47, 2.61]
Gender homophily (vs both male)			
Male recipient, female provider	2.25 [1.24, 4.04]	3.64 [1.30, 10.2]	1.12 [0.71, 2.66]
Female recipient, male provider	1.85 [0.84, 3.98]	0.88 [0.22, 3.46]	1.37 [0.73, 1.72]
Both female	3.21 [1.53, 6.75]	2.20 [0.63, 7.57]	1.22 [0.65, 2.29]

^a^Binary logistic models.

^b^Ordinal logistic models. Values are odds ratios and 95% confidence intervals.

## Discussion

In this rural South African setting, informal care for older persons with care needs was spread widely among family, friends, and neighbors—with limited paid caregiving and support from formal sources. The community nature of rural South Africa might contribute to the wide spread of care; this finding aligns with existing literature suggesting that proximity facilitates more intensive caregiving efforts (Reinhard et al., 2012). Care was most commonly provided by unemployed female relatives one or two generations younger than the recipient. However, a smaller number of spouses and paid caregivers provided the most intensive care. Notably, all paid caregivers were female. One-third of our care recipients had spousal caregivers, with female spouses frequently reporting being primary caregivers; male spouses were rarer, reflecting large marital age gaps, higher female life expectancy (and thus higher mortality among men), and probably also gender norms. This spousal primacy is common globally (Bainbridge et al., 2006; Park et al., 2015), and may reflect strong marital commitment, cultural expectation to care for one’s spouse in times of need (Bohman et al., 2009), or both. In some cases, inheritance, shared assets (e.g., house), and continued access to family support may also have influenced the decision to continue to provide care. This finding supports extensive evidence on the gendered nature of caregiving, where women are more likely to assume caregiving roles and provide more intensive care (Bainbridge et al., 2006; Pinquart & Sörensen, 2003).

Although gender-imbalanced spousal care may reflect available support capacity, greater numbers of female caregivers and more intensive care from female caregivers were consistent across relationship types. These patterns reflect gender norms that expect women to assume primary caregiving roles; such caregiving, in turn, may perpetuate gender inequalities in labor force participation and other opportunities (Roos & Wheeler, 2016; Rutagumirwa et al., 2020). Living arrangements and household structures in South Africa are also influenced by gender and age, which in turn affect the availability of care. Women, particularly daughters and granddaughters, are more likely to provide care than their male counterparts (Agyemang, 2021; Schatz, Seeley, & Zalwango, 2018). Furthermore, gender-inequitable care places particular pressure on female-headed households, which are common in South Africa in part due to high male labor migration, and AIDS- and NCD-related mortality (Nwosu & Ndinda, 2018; Nzima & Maharaj, 2020). This dual role as caregiver and household head can further exacerbate women’s financial struggles and increase their vulnerability to poverty. Notably, the cultural norm of providing care within the family, rather than seeking external or institutional support, further reinforces the gendered nature of caregiving.

Children commonly care for older adults worldwide, reflecting intertemporal reciprocity of care that may be self-interested or altruistic (Silverstein et al., 2002). We found grandchildren and great-grandchildren contributing significantly to caregiving in rural Mpumalanga, with at least three possible, likely overlapping, explanations for the frequency of this “skipped generation” care. First, it may reflect direct reciprocity in a setting where many grandparents raised their grandchildren due to HIV-related mortality, economic migration, or other personal reasons (Adamek et al., 2020; Schatz, Ralston, et al., 2018). Furthermore, the social relationships within these households play a crucial role in caregiving dynamics. Strong kinship ties and a sense of intergenerational obligation often dictate who provides care. This reciprocal relationship is a key aspect of caregiving in rural South African communities (Harling et al., 2020). Second, grandchild care roles may also reflect the financial support older relatives provide to them via the Older Person’s Grant, a means-tested noncontributory monthly pension paid to people aged over 60 years which is often a primary income source in poor South African households (Waidler & Devereux, 2019); in the wider HAALSI survey 84% of over-60-year olds reported receiving such a pension in 2019. Third, grandchild caregiving may reflect opportunity and convenience as youth, unable to find work, live and seek opportunities in rural homes (Honwana, 2014). Granddaughters may have given birth in the area and have elected to stay at their grandparent’s home, combining elder care and infant care in the early years of parenting. Centrally, the multigenerational households prevalent in rural South Africa offer a built-in support system for caregiving, allowing care responsibilities to be shared among family members across generations (Ardington et al., 2010). This is important for ensuring older people receive care in the context of limited resources and social support systems, although it also potentially places financial strain on older people in their households (Schatz, Ralston, et al., 2018).

Although household residents provided more care than nonresidents in our sample, beyond-household informal support was substantial. The preponderance of within-household care reflects both within-family propinquity (i.e., shared history, reciprocity, mutual understanding, and emotional bonds) and pragmatics—small acts of care are easily anticipated and undertaken when caregiver and care recipient are together, and thus care tasks expand where the caregiver and care recipient are proximate (Adedeji et al., 2022; Schatz, Ralston, et al., 2018). Nevertheless, care recipients received care (of varying sorts and time) from an average of five nonco-resident family and around 1.5 nonfamily members. The presence of geographically and socially close-knit communities in rural villages provides access to wider support networks, influencing caregiving practices and leading to resource-sharing such as driving care recipients to clinic (Schatz, Ralston, et al., 2018).

Only 19 care recipients had paid caregivers, but these employees provided the most intensive care of any group. The limited availability of paid care might reflect a desire from relatives to give care themselves—potentially linked to ideas of reciprocity and social exchange within families (Akinrolie et al., 2020; Schatz, Seeley, & Zalwango, 2018). However, it also reflects the very limited resources available within care recipients’ households, many of which depend almost exclusively on government grants—only 20% of respondents had fulltime employment. Although paid care appeared to play a critical role for those households able to afford it, these caregivers were not formally qualified and qualitative work with the cohort suggests they are often paid very poorly (Brear et al., 2024). Our findings align with previous research indicating that caregivers who spend more time providing care tend to develop greater confidence and expertise in managing caregiving responsibilities (Pinquart & Sörensen, 2003; Schulz & Sherwood, 2008). This increased confidence may stem from the repetitive nature of caregiving tasks, which allows caregivers to become more skilled and efficient over time. Additionally, the strong association between tertiary education and higher confidence underscores the importance of educational background in shaping caregivers’ perceptions of their abilities. Higher education levels have been linked to better health literacy and problem-solving skills, which can contribute to greater confidence in managing complex caregiving tasks (Mackenzie et al., 2008; Wolf et al., 2005).

### Strengths and Limitations

Our findings provide an overview of the distribution of caregiving for older people in one rural area of South Africa. The study benefited from its nesting within an existing decade-long cohort providing rich background information on the care recipients, and our response rate was very high. By casting a wide net, our study captured many actual and potential caregivers of older individuals, providing a comprehensive view of their care support network. However, the study also has limitations. Its cross-sectional design means we did not capture the dynamic nature of caregiving over time. Longitudinal studies are needed to examine how caregiving dynamics evolve and affect caregivers’ and care recipients’ well-being, something the linked year-long qualitative data may be able to inform. Our use of self-reported caregiving quantity and primacy may have been affected by systematic over-reporting (men were more likely to report being primary caregivers despite providing fewer hours of care on average than women). Further, breadth of our descriptive analysis limits the depth in any one area—future analyses could identify how the availability of, e.g., paid care affects others’ care provision and wellbeing. Finally, our use of an algorithm rather than clinical assessments to identify care recipients may have led to misidentification of those with greatest care needs. Although this algorithm has not been validated to date, it did identify individuals with substantial care needs, as observed ethnographically (Brear et al., 2024). Future validation is planned as part of the HAALSI Dementia study.

## Conclusion

The distribution of care given to older people in rural South Africa is complex. Care is typically shared amongst a range of household residents, nonresident kin and nonkin. As in high income country settings, caregiving is gendered with women providing more care than men, regardless of age of caregiver. In our setting, grandchildren and great-grandchildren of the care recipient provided as much care as children. Although we have shown how care is shared, the motivations behind this pattern of care, and their dynamics over time, require additional investigation. Such deeper understanding is essential for developing targeted interventions and support systems to address the caregiving needs of vulnerable populations—both care recipients and providers—to promote greater equity in caregiving responsibilities.

## Supplementary Material

gbaf008_suppl_Supplementary_Materials
