# Supplementary material for: Distribution of Informal Caregiving for Older Adults Living With or At Risk of Cognitive Decline Within and Beyond Family in Rural South Africa
Source: J Gerontol B Psychol Sci Soc Sci. 2025 Jan 25;80(5):gbaf008. doi: 10.1093/geronb/gbaf008 (PMC11974393; doi:10.1093/geronb/gbaf008)
Supplement: gbaf008_suppl_Supplementary_Materials [file gbaf008_suppl_supplementary_materials.docx]

***The Journals of Gerontology, Series B: Psychological Sciences and Social Sciences* Supplementary Material: Matina et al. Distribution of informal caregiving for older adults living with or at risk of cognitive decline within and beyond family in rural South Africa.**

Supplementary Table 1: Care hours provided by relationship status

|  | Mean ± SD  hours per provider | Mean [SD]  hours per recipient |
| --- | --- | --- |
| Spouse | 18.1 ± 13.2 | 5.5 ± 11.1 |
| Non-spouse peer generation or above | 4.1 ± 7.6 | 3.7 ± 8.6 |
| Children | 5.1 ± 7.0 | 13.9 ± 16.5 |
| Non-child child generation | 4.1 ± 6.1 | 5.3 ± 9.2 |
| Grandchild and beyond | 5.6 ± 8.5 | 16.6 ± 22.6 |
| Friends | 4.4 ± 4.6 | 1.9 ± 4.5 |
| Neighbor | 2.8 ± 3.6 | 2.1 ± 7.7 |
| Employee | 21.9 ± 17.1 | 3.9 ± 11.4 |
| All caregivers | 5.5 ± 8.4 | 52.8 ± 38.2 |

Notes. IQR: Interquartile range; SD: standard deviation. Hours per provider refers to average hours among all caregivers with a given relationship status. Hours per recipient refers to all hours by all caregivers with a given relationship status divided by 106 care recipients.

Supplementary Table 2: Distribution of dependent caregiving variables

| Characteristic | Overall N = 1,012 | Male N = 397 | Female N = 615 | p-value |
| --- | --- | --- | --- | --- |
| Primary caregiver |  |  |  |  |
| by care hours, % | 11.9% | 5.8% | 15.8% | <0.001 |
| self-reported, % | 29.2% | 22.5% | 33.4% | <0.001 |
| Weekly hours of care, Median (IQR) | 2 (1, 7) | 2 (0, 5) | 3 (1, 8) | <0.001 |
| Proportion of care, Median (IQR) | 0.06 (0.02, 0.14) | 0.05 (0.00, 0.10) | 0.07 (0.03, 0.16) | <0.001 |
| Confidence in ability to care ^a^ |  |  |  | 0.80 |
| Not at all confident | 4.5% | 5.2% | 4.0% |  |
| A little confident | 20.8% | 20.6% | 21.0% |  |
| Somewhat confident | 17.1% | 16.0% | 17.8% |  |
| Mostly confident | 37.2% | 36.2% | 37.8% |  |
| Extremely confident | 20.3% | 22.0% | 19.4% |  |

Notes. IQR: Interquartile range. Gender differences assessed by Wilcoxon rank sum test for continuous variables and Pearson's Chi-squared test for categorical ones.

^a^ Only those who reported provided care in the last week were asked about capacity to provide care; overall N = 782, male N = 287, female N = 495.

Supplementary Table 3: Multilevel regression models to predict caregiving per provider as a proportion of all caregiving

|  | Percentage of care | Difference in mean marginal hours |
| --- | --- | --- |
| Co-resident vs non-resident | 1.77 [1.45, 2.09] | 6.34[ 4.09; 8.59] |
| Caregiver age (vs ≥ 60) |  |  |
| < 18 | 1.03 [0.46, 1.60] | 0.15[ -5.21; 5.51] |
| 19-39 | 1.07 [0.57, 1.56] | 0.42[ -3.90; 4.73] |
| 40-59 | 1.12 [0.68, 1.55] | 1.06[ -2.39; 4.50] |
| Marital status (vs Married) |  |  |
| Never married | 1.03 [0.80, 1.27] | 0.51[ -1.85; 2.87] |
| Previously married | 0.97 [0.71, 1.22] | -0.28[ -2.58; 2.01] |
| Work status (vs full-time work) |  |  |
| Part-time work | 1.45 [1.01, 1.90] | 4.37[ 0.17; 8.57] |
| Seeking work | 2.05 [1.44, 2.66] | 9.34[ 5.27; 13.4] |
| Out of workforce | 1.38 [0.98, 1.79] | 3.30[ 0.53; 6.07] |
| Gender homophily (vs Both male) |  |  |
| Male recipient, female provider | 1.20 [0.98, 1.42] | 2.09 [ -0.43; 4.61] |
| Female recipient, Male provider | 0.94 [0.71, 1.16] | -0.60[ -3.14; 1.94] |
| Both female | 1.55 [1.16, 1.94] | 5.17[ 2.10; 8.25] |
| Age difference (per decade) | 0.90 [0.82, 0.98] | -1.04[-1.85; -0.23] |
| Relationship type (vs Spouse) |  |  |
| Non-spouse peer generation or above | 0.33 [0.17, 0.49] | -8.13[-11.17; -5.09] |
| Child | 0.44 [0.22, 0.67] | -7.97[-13.09; -2.85] |
| Non-child child generation | 0.44 [0.22, 0.67] | -6.83[-10.58; -3.07] |
| Grandchild and beyond | 0.53 [0.25, 0.81] | -6.41[-12.02; -0.79] |
| Friends | 0.35 [0.18, 0.52] | -7.35[-10.19; -4.50] |
| Neighbor | 0.25 [0.12, 0.38] | -8.76[-11.30; -6.23] |
| Employee | 1.86 [0.82, 2.90] | 8.64[ -5.05; 22.32] |
| Education (vs Primary) |  |  |
| No Education | 0.93 [0.64, 1.22] | -0.55[ -3.46; 2.36] |
| Secondary Education | 0.81 [0.62, 0.99] | -2.24[ -4.61; 0.12] |
| Tertiary Education | 0.55 [0.34, 0.75] | -4.85[ -6.96; -2.73] |
| Care recipient predicted dementia (vs no dementia) |  |  |
| Mild dementia | 0.85 [0.67, 1.03] | -1.74[ -4.33; 0.85] |
| Moderate to severe dementia | 0.96 [0.67, 1.26] | -0.06[ -3.24; 3.11] |


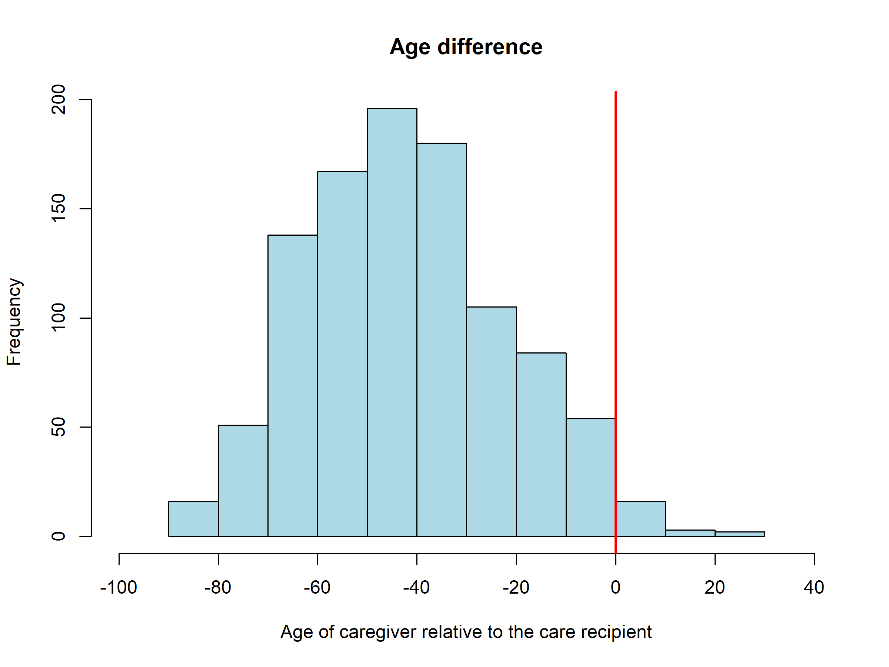


Supplementary Figure 1: Age difference between care recipient and caregiver (negative values mean caregiver is younger)


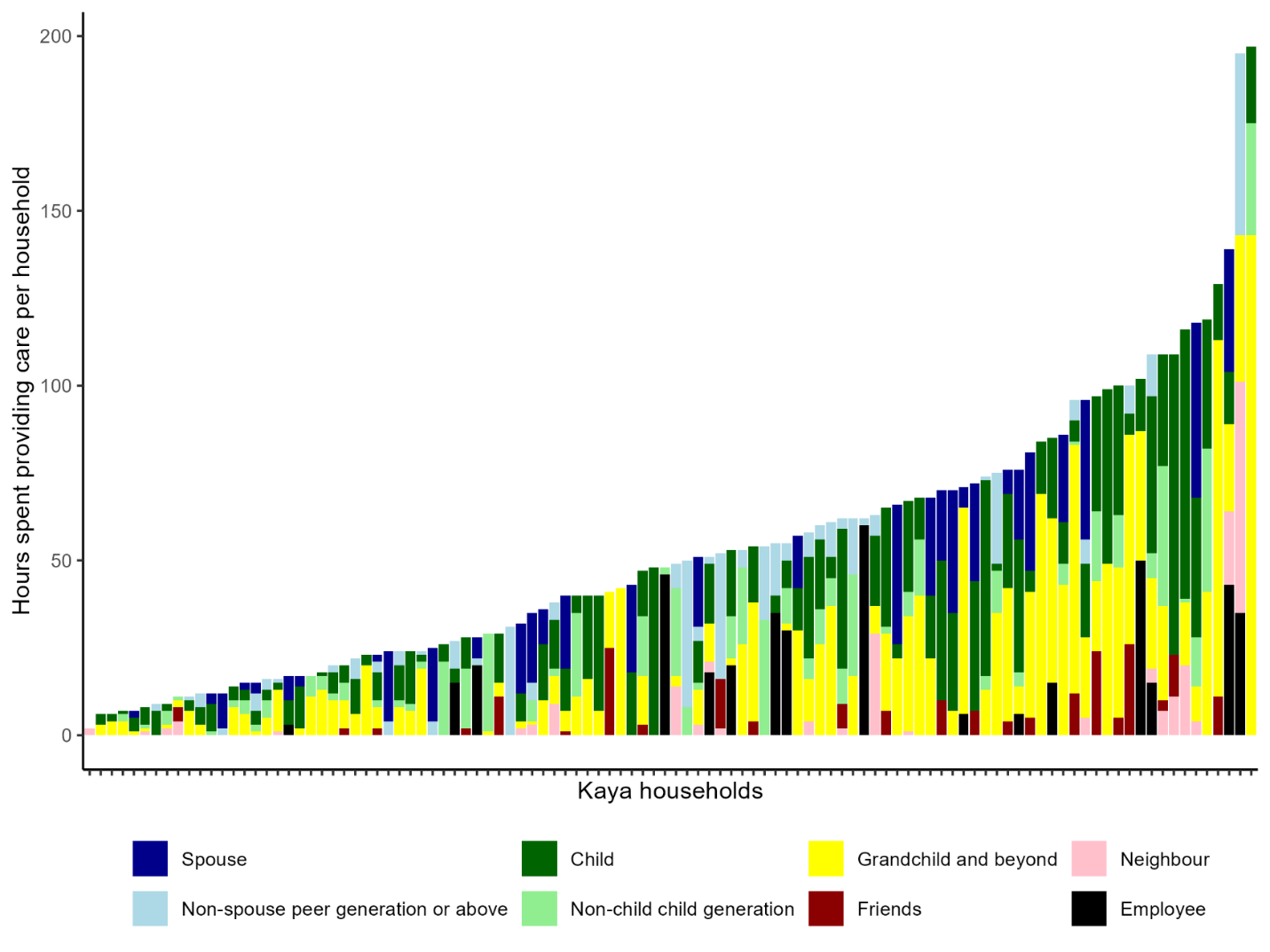


Supplementary Figure 2: Composition of hours of care provided for each care recipient by relationship type


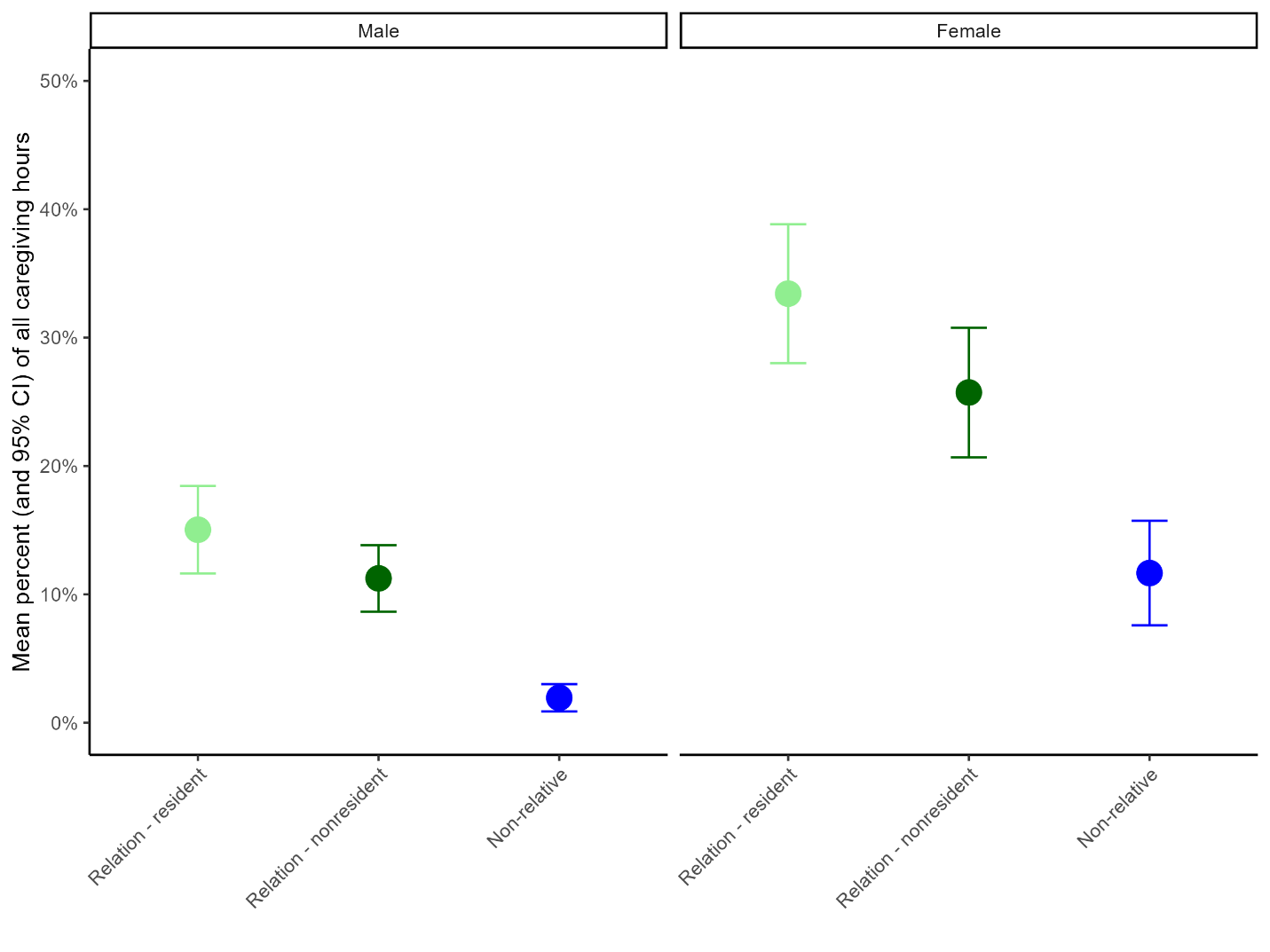


Supplementary Figure 3: Distribution of caregiver hours from care recipient's point of view based on household residency status and gender
